# Effect of Core Architecture on Charpy Impact and Compression Properties of Tufted Sandwich Structural Composites

**DOI:** 10.3390/polym13101665

**Published:** 2021-05-20

**Authors:** Chen Chen, Peng Wang, Xavier Legrand

**Affiliations:** 1 University of Lille, Ensait, Gemtex, F-59000 Roubaix, France; chen.chen@ensait.fr (C.C.); xavier.legrand@ensait.fr (X.L.); 2 University of Haute-Alsace, Ensisa, Lpmt, F-68000 Mulhouse, France

**Keywords:** textile composites, sandwich, tufting, compression, Charpy impact

## Abstract

This study presents a novel sandwich structure that replaces the polypropylene (PP) foam core with a carbon fiber non-woven material in the tufting process and the liquid resin infusion (LRI) process. An experimental investigation was conducted into the flatwise compression properties and Charpy impact resistance of sandwich composites. The obtained results validate an enhancement to the mechanical properties due to the non-woven core and tufting yarns. Compared to samples with a pure foam core and samples without tufting threads, the compressive strength increased by 45% and 86%, respectively. The sample with a non-woven layer and tufting yarns had the highest Charpy absorbed energy (23.85 Kj/m^2^), which is approximately 66% higher than the samples without a non-woven layer and 90% higher than the samples without tufting yarns. Due to the buckling of the resin cylinders in the Z-direction that occurred in all of the different sandwich samples during the compression test, the classical buckling theory was adopted to analyze the differences between the results. The specific properties of the weight gains are discussed in this paper. The results show that the core layers have a negative effect on impact resistance. Nevertheless, the addition of tufting yarns presents an obvious benefit to all of the specific properties.

## 1. Introduction

Sandwich composites normally consist of two face sheets with high in-plane mechanical properties and a lightweight core that undergoes shear loading and through-the-thickness (TT) compressive loading. They are applied in many industries, such as the construction, automotive, military, and aerospace industries, due to their advantage in weight saving [1]. At the request of being light-weight, a conventional sandwich usually uses polymer foam as the core material [2]. However, a sandwich with low-density core materials usually presents weaknesses in the TT direction, including low impact resistance and low flatwise compressive strength [3]. In addition, a common problem of the sandwich structure is the delamination between face sheets and core materials [4].

Therefore, it is necessary to improve the capacity to bear TT loading. The most common method is inserting a TT reinforcement, which can be achieved with Z-pins [5,6,7,8,9], 3D weaving [10,11,12], stitching [13,14,15], and tufting [16,17,18]. Additionally, a TT reinforcement can augment shear resistance to prevent delamination [13]. Some studies present experimental and modeling investigations on a 3D sandwich. Che et al. compared the compression performance of an octahedrally stitched sandwich composite with a sandwich that had cellular core materials [19]. May-Pat et al. used the conventional relations of equivalent series-parallel springs to estimate the compressive and shear properties of sandwiches and analyzed the weight gain caused by the massive absorbed resin [20]. Jijun et al. [21] fabricated a novel X-Truss/foam sandwich structure with Z-pins inserted and bias stitching, and the compression test was conducted to evaluate these two methods. The results indicated that the specimens made from the Z-pin process have a higher compression modulus compared to those made with bias stitching. The mechanical properties of a foam core with perforations and stitching were investigated by Yalkin et al. [22], and they found that the newly proposed stitched core specimens with a relatively insignificant weight increase have superior mechanical performance compared to plain core specimens. Daowu analyzed the effects of the initial curvature and the angle of inclination of core fibers on the TT elastic moduli and compressive strength of the fibrous core [23]. Long et al. investigated the influence of the sandwich thickness, TT fiber density, and other parameters on compression behavior [24]. Cartié and Fleck modeled the buckling load and compressive strength of Z-pins [25]. Mouritz and Nanayakkara improved the calculation model of compressive strength and modulus based on these studies [2,3]. Nishi et al. [26] investigated the Charpy impact property of sandwiches with a polycarbonate core. Srivastava analyzed the impact behaviors of sandwiches with a polyurethane foam core and E-glass fiber face sheets with the Charpy impact test, the Izod impact test, and the weight drop test [27].

However, the research objects of these works are usually a sandwich structure with a pure foam core; different core materials are not considered. Changing the design and raw materials of the core construction can affect both the compressive and impact properties of the sandwich composite [28]. In addition to TT reinforcement, another method to improve load capacity is to adopt core materials with higher mechanical properties. Non-woven fabrics are considered a potential replacement.

The originality of this study is the introduction of both non-woven core material and TT tufting yarns in a sandwich structure to improve its mechanical performance. The influence of these two reinforcements is determined using compression and Charpy impact experimental investigation.

## 2. Materials and Methods

### 2.1. Process of Sandwich Composite Manufacturing

**Figure 1:**
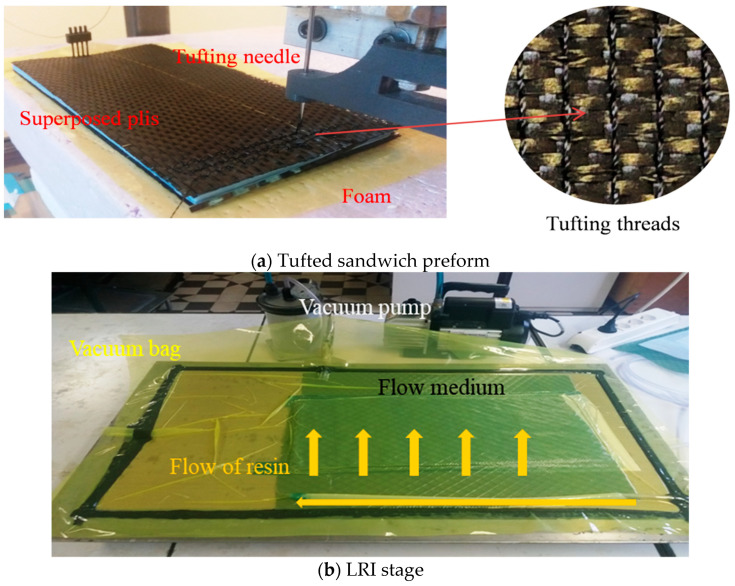
Manufacturing process of the sandwich composite.

To insert the reinforcements in Z-direction, a tufting needle with yarn inside punctured the superposed layers. The yarn was retained within the preform when the needle retracted (see Figure 1). After the sandwich preform was produced, it could be impregnated using the LRI process, where the voids in an evacuated preform were filled with a liquid epoxy resin. When the resin solidified, the solid resin bound all the raw materials of the sandwich into a unified rigid composite.

**Figure 2:**
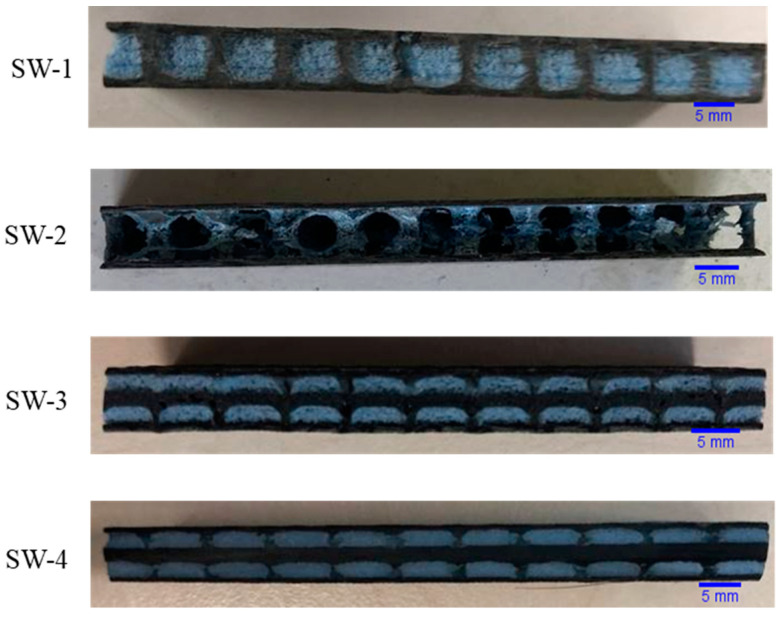
Photos of different sandwich structures.

Four groups with different core architectures were prepared (shown in Table 1). The sandwich core could be divided into two parts: the TT resin cylinders parallel to the Z-direction and the core layers normal to the Z-direction. All of the sample sets were reinforced by tufting with yarns except SW-4, which was punctured by a needle without yarn. As a result, the TT column of SW-4 was pure resin, while the columns of the other groups consisted of carbon fiber and resin. The superposed core layers of SW-1 were the same as SW-2: three plies of foam. The difference was that the foam of SW-2 was later removed, leaving only the columns connected by the two skins. In SW-3 and SW-4, the central foam layer was replaced by a non-woven mat (see Figure 2).

The basic information about the raw textile materials, the foam, and the resin are presented in Table 2.

### 2.2. Experimental Methods

#### 2.2.1. Flatwise CompressiveTtest

**Figure 3:**
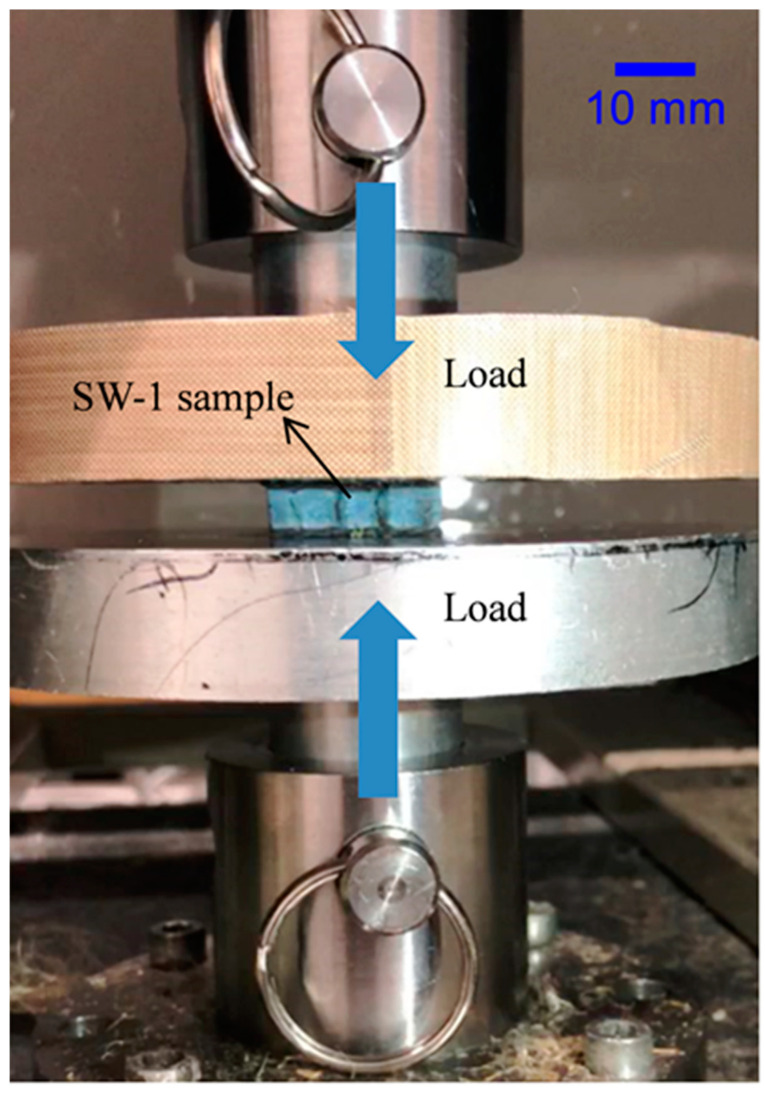
Compressive test device.

To determine the compressive strength in the Z-direction, where the core would be placed, in structural construction, the test was designed according to the standard ASTM C365-16 [29]. The general principle of the compression tests is shown in Figure 3. The specimens were parallelepipeds with a square base with a dimension of 25 × 25 mm^2^. The specimens to be tested needed to be placed exactly in the center of the head of the indenter. Each test was repeated five times to ensure high repeatability of the testing and results. The flatwise compressive strength (*σ_z_*) and its specific value (*σ_zs_*) are given by the following:

(1) $$\sigma_{z}=P/b^{2}$$

(2) $$\sigma_{zs}=P/\rho b^{2}$$

where *P* is the measured value of the load, *b* is the width of the sample, and ρ is the density of the sample. The compressive modulus (*E_z_*) and the specific modulus (*E_zs_*) are determined from the stress–strain curves obtained using the following equation:

(3) $$E_{z}=\sigma_{Z}\times t/d$$

(4) $$E_{zs}=\sigma_{Z}\times t/\rho d$$

where *t* is the thickness of the sandwich specimen and *d* is the displacement of the moving loading plate.

#### 2.2.2. Charpy Impact Test

**Figure 4:**
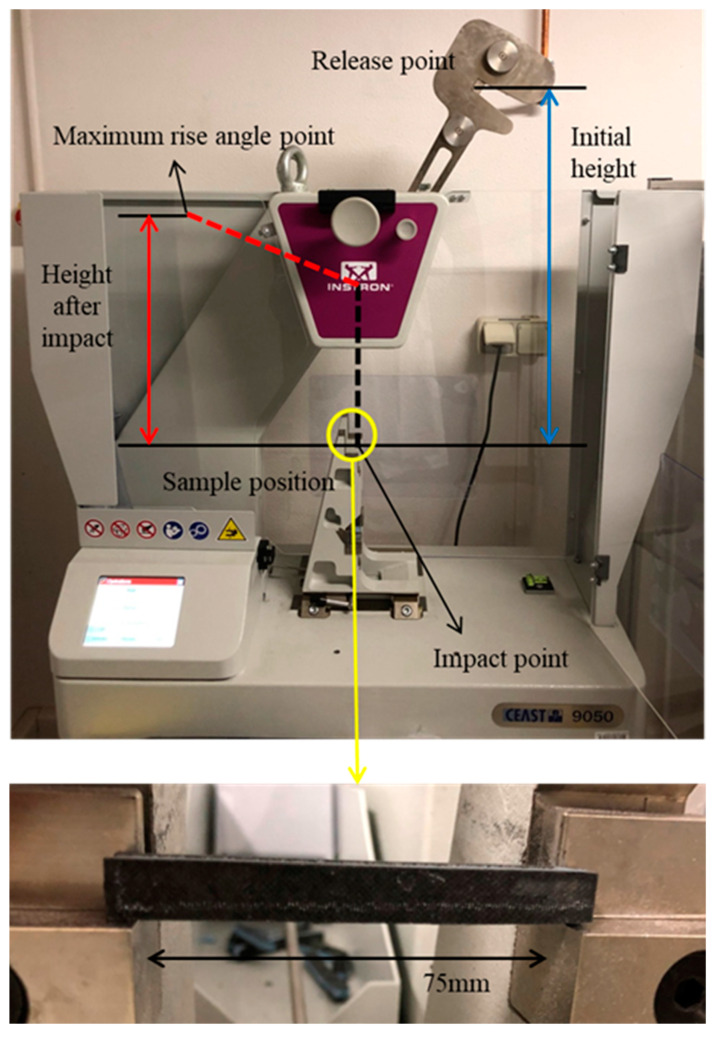
Charpy impact test schematization.

For sandwich composites, usually, only tests in the flatwise direction are carried out. The Charpy impact strength of the composites was tested according to EN ISO 179-1 [30]. This test was designed to measure the resistance to failure of a material subjected to a suddenly applied impact. The value measured was the impact energy or the energy absorbed before fracture. The apparatus consisted of a pendulum of known mass and length that was dropped from a known height in order to impact a notched specimen of material. The energy transferred to the material could be inferred by comparing the difference in height of the hammer before and after the fracture (energy absorbed by the fracture event, see Figure 4), which is denoted by *W*. The length (*l*) and width (*b*) of the specimens were 80 mm and 10 mm, respectively. The resilience (*K*) can be computed by the following:

(5) K=W/lb

Considering the mass of samples, the specific resilience is denoted by *K_S_* and is determined by the following:

(6) $$K_{s}=K/\rho$$

## 3. Results

### 3.1. Compression

**Figure 5:**
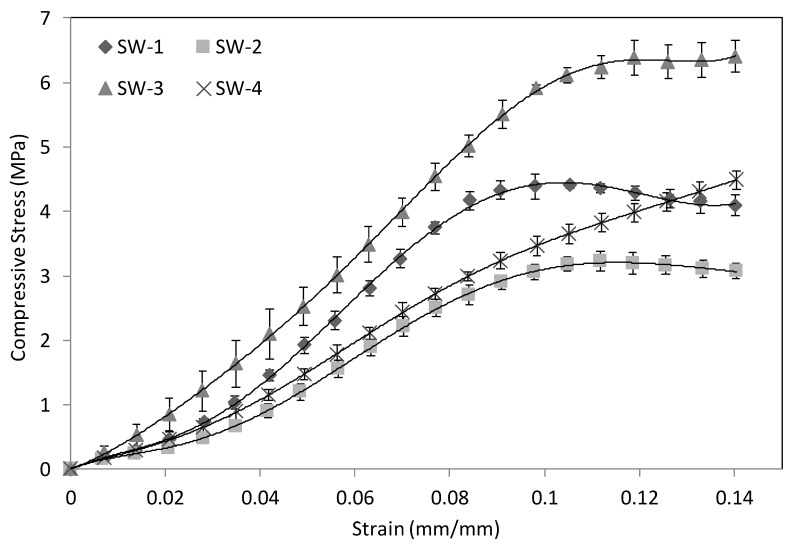
Flatwise compression stress–strain curves.

The effects of the core materials and the tufting yarns on the resistance to flatwise compression loading are shown in Figure 5. The compressive stress–strain curves of the sandwich samples with four different cores are presented. The curves show an initial elastic stage in which the slope represents the compressive modulus. The maximum stress values before failure of the samples are arranged in descending order: 6.40 MPa in SW-3, 4.42 MPa in SW-1, 3.45 MPa in SW-4, and 3.24 MPa in SW-2 (see Table 3).

The compressive load was mainly carried by the core materials; thus, the analysis of the skins does not have any importance. After the first peak stress, the resin columns buckled or fractured and stress began to decrease. However, the core layers resisted the compression and increased the stress as the compression proceeded because of densification [3,31]. Thus, only the structural failure phase (the first peak stress) was analyzed. The behavior of a sandwich tufted with yarns (SW-1, SW-2, and SW-3) was different from that of a sandwich without tufting threads (SW-4). The curves of SW-1, SW-2, and SW-3 show a small drop following the ultimate compression stress due to the breaking of the composite columns. On the contrary, the stress value of SW-4 has no downward trend after the failure point and keeps increasing, and only the slope becomes smaller. Although SW-3 generally experienced a larger load drop than SW-4 following the failure point, SW-3 presented a higher compressive load-bearing capacity in the entire strain range. The profits from the yarns in Z-direction is clear compared to the sandwich panels with no tufting threads, which can be explained by comparing Equations (11) and (12). The difference is determined by the modulus and the volume fraction in the TT columns with tufting yarns.

The effects of the foam core on the ultimate stress until failure and the compressive modulus can be obtained by comparing SW-1 and SW-2. The stress value and the slope of SW-1 are higher, which indicates that compressive strength and modulus increase with the presence of a foam core. The columns in the Z-direction and the core layers (foam or foam/non-woven) carry the compressive load together. Nevertheless, by using Equations (9)–(11) and (13), it can be seen that the increases in the stress value of non-woven materials are higher than those of pure foam.

Considering the weight increase caused by the non-woven layer, the comparison of specific stress is more significant. The results in Table 3 show that SW-1 and SW-3 have higher values, which are 9.52 MPa and 9.27 MPa, respectively. These two values are very close. Taking into account the coefficient of variation, it can be considered that the specific compressive properties of these two groups are the same. The reason for the increase in mass is that the higher porosity and hygroscopicity of the non-woven material itself causes the absorption of excessive resin [32,33].

Though the external observation of the failure mode was not carried out during the compression process due to either the foam or non-woven materials covering the deformation of the columns in the core, the fracture of the columns was confirmed after the test. These ruptures occurred near the junctions between the skin and the columns. The foam was critically flattened, and it could not recover its original thickness, but the non-woven core remained the same as it had been initially. The bulking and the cracking of the foam and the breaking of the columns are the principal failure modes. The longitudinal splitting of the columns under the compressive load followed by its deformation caused the foam to crack and collapse, which led to a large loss of the entire core’s rigidity.

**Figure 6:**
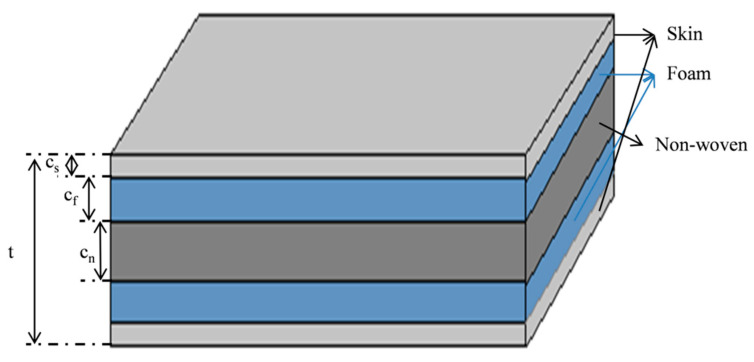
Schematic for calculation of the SW-3 and SW-4 samples.

In the case of SW-2, only the fiber/resin columns underwent compressive loading. Thus, the failure mode is the buckling of the columns, and the failure load (*F*_*sw*2_) was reached when the columns reached their critical buckling load.

(7) $$F_{sw2}=\pi^{2}E_{col}I/{\left(Kt\right)}^{2}$$

where *E_col_* is the elastic modulus of columns, *I* is the area moment of inertia, and *K* is the column effective length factor that depends on the conditions of the end support of the column. As both ends of the column are considered fixed, the value of *K* is 1 in this paper. Consequently, the equation of the column’s compressive strength that fails by buckling can be presented as follows:

(8) $$\sigma_{col}=\pi^{2}r^{2}E_{col}/4t^{2}$$

where *r* is the radius of the TT columns. The compressive stress (*σ_z_*) can be predicted by the following:

(9) $$\sigma_{z}^{sw1}=\pi^{2}r^{2}E_{col}\upsilon_{col}/4t^{2}+E_{f}\varepsilon \upsilon_{f}$$

(10) $$\sigma_{z}^{sw2}=\pi^{2}r^{2}E_{col}\upsilon_{col}/4t^{2}$$

(11) $$\sigma_{z}^{sw3}=\pi^{2}r^{2}E_{col}\upsilon_{col}/4{\left(Kt\right)}^{2}+E_{c}\varepsilon \left(1-\upsilon_{col}\right)$$

(12) $$\sigma_{z}^{sw4}=\pi^{2}r^{2}E_{r}\upsilon_{col}/4{\left(Kt\right)}^{2}+E_{c}\varepsilon \left(1-\upsilon_{col}\right)$$

where the superscripts *sw*1, *sw*2, *sw*3, and *sw*4 represent the four different groups; *ε* is the compressive strain; *E* is Young’s modulus; and the subscripts *r* and *f* represent the resin and the foam, respectively. Young’s modulus of the core with non-woven layers (*E_c_*) is obtained by using the following [20]:

(13) $$E_{c}=\left(2c_{f}+c_{n}\right)E_{f}E_{n}/\left(2c_{f}E_{n}+c_{n}E_{f}\right)$$

where *c* is the thickness and the subscripts *n* represent the non-woven material (see Figure 6).

### 3.2. Charpy Impact

**Figure 7:**
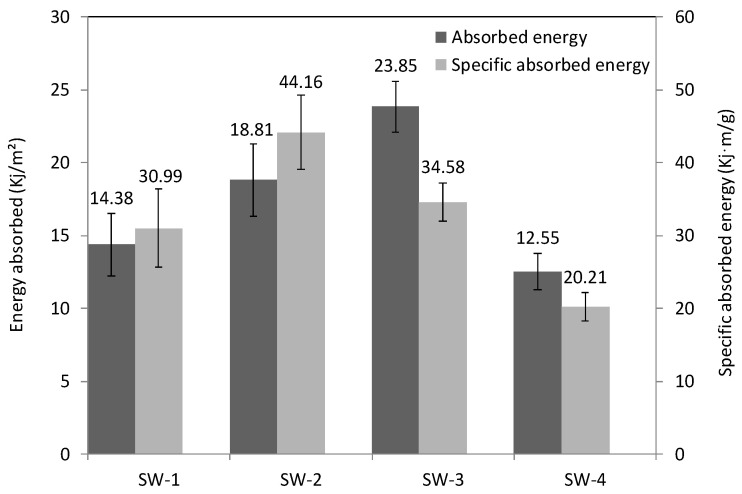
Impact energy absorbed during the Charpy test.

**Figure 8:**
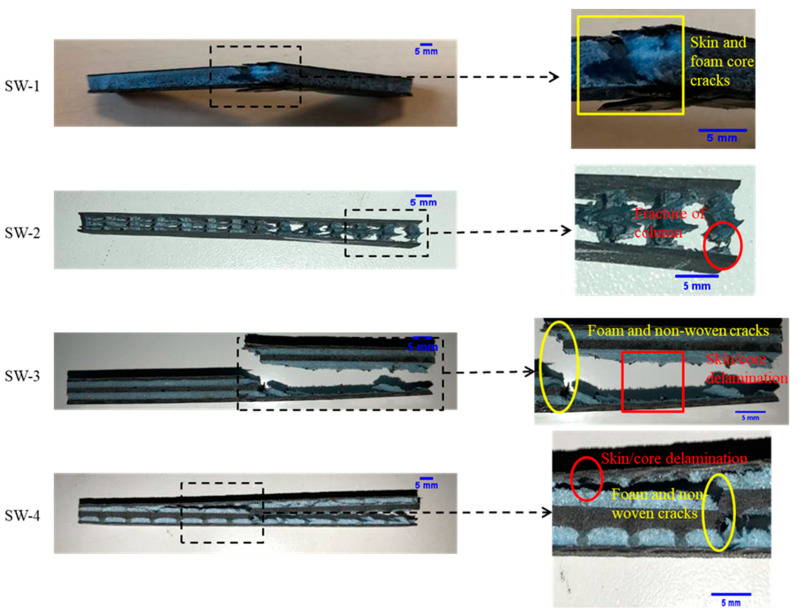
Fractured specimens after the Charpy impact test.

The results of the Charpy absorbed energy (Kj/m^2^) of each group are presented in Figure 7. SW-3 has the highest impact strength (23.85 Kj/m^2^) among these four groups of samples. PP foam has a low fracture toughness; it promoted the extension of cracks from the top skin to the bottom skin by connecting these two skins. This resulted in a rupture of both skins (as shown in Figure 8) and a lower absorbed impact energy. Compared with the hollow structure (SW-2), the presence of the foam core (SW-1) increased the brittleness of the core. The absorbed energy of SW-3 is approximately 66% higher than that of SW-1 (14.38 Kj/m^2^). Pure epoxy resin and carbon fiber are normally considered brittle materials [34]. However, toughness is related to both strength and brittleness. The tensile strength and elongation at the break of the resin-infused non-woven composite were measured as 63.79MPa and 1.49%, respectively. Compared with the parameters the of foam (1.02 MPa and 6.7%), it can be obtained that the non-woven composite layer has a higher toughness. This can contribute to improving the toughness of the whole sandwich structure. Moreover, the higher absorbed energy of the fiber/resin columns compared with the pure resin columns (SW-4) can be explained by the same mechanism.

Although the presence of carbon fiber materials can increase the absorbed energy, it also increases the whole sandwich mass, which goes against the lightweight design trend. Thus, the introduction of the notion of specific absorbed energy is significant. The results indicate that the SW-2 with a hollow structure presents the largest value, as it is the lightest, and that it has a relatively high absorbed energy.

The photographs of the fractured samples of each set are presented in Figure 8. A brittle fracture tendency can be seen in the sandwich with core layers (SW-1, SW-3, and SW-4) by visual analysis, which shows more serious damage to the core structure. The delamination between the core and the skin can be observed in all of the sandwich groups except SW-1, and the cracks of the foam and non-woven layer are determined. The fracturing of the columns mainly occurred at the connection points between the columns and the composite layers (the fabric skin and the non-woven core ply), which is the weak zone of the columns’ mechanical property.

## 4. Conclusions

Tufting yarn in the Z-direction and the non-woven core layer can effectively improve flatwise compressive properties and Charpy impact tenacity by increasing the compression modulus, strength, and absorbed strain energy capacity. The ultimate compressive strength is determined by the elastic modulus of the TT columns, both foam and non-woven. In addition, the volume fraction of the columns and non-woven material also plays an important role. The columns’ reinforcements failed due to buckling. However, the specific impact properties were not positively influenced by the presence of tufting yarn and non-woven material. The increase in the sample weight by adding foam or non-woven material is more significant compared to improved absorbed energy, which results in a lower specific impact property.

The manufacturing process and the sandwich core structure do not allow for an accurate calculation for the parameters of columns and the non-woven core material; hence, the existing models can only predict the mechanical property’s tendency to augment or diminish. The following work needs to be conducted in the future:

1. Improvement of the manufacturing process to strictly control the resin content of the sample.
2. A new observation method to determine the status of each reinforcement and the failure mode.
3. Improvement of the calculation model to accurately determine the compressive strength and modulus.

## Figures and Tables

**Table 1:** Essential parameters of the samples.

Sample ID	Core Material	TT Reinforcement	Thickness (mm)
SW-1	3 layers of foam	Yarn/resin cylinders	7.27 ± 0.29
SW-2	Hollow		6.75 ± 0.16
SW-3	Foam/Non-woven		6.58 ± 0.17
SW-4		Pure resin cylinders	6.46 ± 0.15

**Table 2:** Parameters of the raw materials.

Raw Materials	Weight Parameter	Young’s Modulus (GPa)	Elongation at Break
Skin fabric	285 g/m^2^	24.4	3.5%
Non-woven	210 g/m^2^	15.8	-
Tufting yarn	Linear density: 2 × 67 Tex	240.0	1.7%
Foam	Density: 0.01 g/cm^3^	1.6 × 10^−2^	6.7%
Epoxy resin	Density: 1.2 g/cm^3^	2.9	1.7%

**Table 3:** Compressive properties of sandwich composites.

Sample ID	Compressive Strength (MPa)	Specific Compressive Strength (kN·m/kg)	CV (Coefficient of Variation)	Compressive Modulus (MPa)	Specific Compressive Modulus (kN·m/kg)
SW-1	4.42	9.52	2.78%	64.32	138.61
SW-2	3.24	7.60	5.48%	45.17	95.70
SW-3	6.40	9.27	3.40%	67.23	97.46
SW-4	3.45	5.55	6.09%	44.82	72.17

## Data Availability

Not applicable.
